# DNA Aptamers against Exon v10 of CD44 Inhibit Breast Cancer Cell Migration

**DOI:** 10.1371/journal.pone.0088712

**Published:** 2014-02-19

**Authors:** Joji Iida, Rebecca Clancy, Jesse Dorchak, Richard I. Somiari, Stella Somiari, Mary Lou Cutler, Richard J. Mural, Craig D. Shriver

**Affiliations:** 1 Department of Cell Biology, Windber Research Institute, Windber, Pennsylvania, United States of America; 2 ITSI-Biosciences, Johnstown, Pennsylvania, United States of America; 3 Department of Pathology, Uniformed Services University of the Health Science, Bethesda, Maryland, United States of America; 4 Windber Research Institute, Windber, Pennsylvania, United States of America; 5 Department of Surgery, Walter-Reed Army Medical Center, Bethesda, Maryland, United States of America; University of Illinois at Chicago, United States of America

## Abstract

CD44 adhesion molecules are expressed in many breast cancer cells and have been demonstrated to play a key role in regulating malignant phenotypes such as growth, migration, and invasion. CD44 is an integral transmembrane protein encoded by a single 20-exon gene. The diversity of the biological functions of CD44 is the result of the various splicing variants of these exons. Previous studies suggest that exon v10 of CD44 plays a key role in promoting cancer invasion and metastasis, however, the molecular mechanisms are not clear. Given the fact that exon v10 is in the ectodomain of CD44, we hypothesized that CD44 forms a molecular complex with other cell surface molecules through exon v10 in order to promote migration of breast cancer cells. In order to test this hypothesis, we selected DNA aptamers that specifically bound to CD44 exon v10 using Systematic Evolution of Ligands by Exponential Enrichment (SELEX). We selected aptamers that inhibited migration of breast cancer cells. Co-immunoprecipitation studies demonstrated that EphA2 was co-precipitated with CD44. Pull-down studies demonstrated that recombinant CD44 exon v10 bound to EphA2 and more importantly aptamers that inhibited migration also prevented the binding of EphA2 to exon v10. These results suggest that CD44 forms a molecular complex with EphA2 on the breast cancer cell surface and this complex plays a key role in enhancing breast cancer migration. These results provide insight not only for characterizing mechanisms of breast cancer migration but also for developing target-specific therapy for breast cancers and possibly other cancer types expressing CD44 exon v10.

## Introduction

Enhanced migration into the surrounding tissues is one of the hallmarks of the malignancy of tumor cells. To successfully metastasize, a cancer cell has to detach from the primary tumor, invade into surrounding tissues, and intravasate into blood or lymphatic vessels. These processes are composed of complex mechanisms involving tumor recognition and degradation of extracellular matrix (ECM) proteins and migration into tissue. In order to develop effective therapeutic strategies for breast cancer, it is important to characterize mechanisms of tumor-ECM interaction.

Agents able to bind tightly and selectively to disease markers can greatly benefit disease diagnosis and therapy. Aptamers are functional molecules, usually DNA or RNA oligonucleotides, with the appropriate sequence and structure that allow them to form a complex with a target molecule. Aptamers are evolved by an iterative selection method called SELEX (*s*ystematic *e*volution of ligands by *ex*ponential enrichment) to specifically recognize and tightly bind their targets by means of well-defined complementary three-dimensional structures [1], [2]. They have been developed against intracellular and extracellular molecules expressed in cancer cells and demonstrated to attenuate the biological functions of their target molecules [3], [4], [5], [6], [7], [8], [9], [10]. Furthermore, DNA aptamers developed against MUC1 have been applied as tools for diagnosis of epithelial tumors as well as imaging of tumor cells in vivo [1], [11], [12], [13]. Thus, aptamers have a great impact for the next generation of strategies for cancer detection, diagnosis, and therapy. Indeed, aptamers against VEGF165, Pegaptanib (brand name Macugen), is an effective therapy for age-related macular degeneration (AMD) [14], [15], [16]. Moreover, Phase II trial of AS1411, aptamer targeting nucleolin, demonstrated its efficacy in renal cell carcinoma patients [17], suggesting that DNA aptamers represent a novel therapeutic strategy for cancer therapy.

CD44 is an integral transmembrane protein encoded by a single 20-exon gene. In the standard form (CD44s), 10 of the 20 exons are translated. Multiple variant isoforms exist (CD44v1-10) which arise from alternate mRNA splicing of the remaining 10 exons [18]. In contrast to the ubiquitous expression of CD44s, splice variants are highly restricted in their expression in normal or malignant tissues. Indeed, previous studies demonstrated that exon v10 plays a role in promoting breast tumor development and progression [19], [20]. High-level expression of this exon, v10, was also reported in malignant renal cell carcinoma cells [21]. Although the mechanisms by which exon v10 facilitates these processes are not entirely clear, it is possible that this exon stimulates migration, adhesion, and growth in microenvironments through mediating the interaction with ECM directly or indirectly by forming molecular complexes with other cell surface molecules. Importantly, previous studies using peripheral blood stem cells suggest that exon v10 plays a key role in promoting leukocytes adhesion to bone marrow cells and migration to disease sites in mice [22], [23], [24]. Thus, as an analogy, it is plausible to suspect that exon v10 of CD44 expressed on cancer cells could play a key role in promoting metastasis by possibly mediating cell adhesion and migration at secondary sites. Therefore, the development of small molecules (i.e. DNA aptamer) that specifically recognize and antagonize the biological functions of exon v10 of CD44 would be a promising approach for treatment of TN breast cancer patients.

In this study, we utilized the SELEX approach to develop DNA aptamers that specifically recognize CD44 exon v10. These aptamers significantly inhibited migration of breast cancer cells. Pull-down assays suggest that the exon interacted with EphA2 and the interaction was inhibited by the aptamers. Given the fact that EphA2 plays a key role in promoting tumor invasion and metastasis, our results suggest a novel model of a molecular complex consisting of CD44 and EphA2 that facilitates TN breast cancer progression.

## Materials and Methods

### Cell Lines

All cell lines used in this study (HCC38, SK-Br3, MCF-7, MDA-MB-231, and HCC1806) were purchased from ATCC and maintained in RPMI1640 supplemented with 10% FBS.

### Antibodies and Chemicals

MOPC-21 (IgG_1_), pFLAG-CTC vector, and Streptavidin-FITC were purchased from Sigma-Aldrich (St. Louis, MO, USA). Anti-CD44 (clone 156-3C11) and anti-α2 integrin subunit (clone P1E6, IgG1) antibodies were purchased from Millipore (Billerica, MA, USA). FITC-conjugated goat anti-mouse IgG, FITC-conjugated goat anti-rabbit IgG, HRP-conjugated rabbit anti-mouse IgG, HRP-conjugated rat anti-rabbit IgG, and HRP-conjugated Streptavidin were purchased from Jackson Immunoresearch (West Glove, PA, USA). Anti-CD44v10 antibody (AB2082) was purchased from Calbiochem (San Diego, CA, USA). Anti-FLAG (M2) antibody was purchased from Stratagene (Santa Clara, CA, USA). Type I Collagen was purchased from Inamed Biomatenak (Freemont, CA, USA). Enhanced chemiluminescence (ECL) reagents were purchased from GE Healthcare (Piscataway, NJ, USA). Other chemicals were purchased from Sigma-Aldrich unless otherwise mentioned.

### Migration Assays

Migratory abilities of breast cancer cells were evaluated using 24-well Transwell systems (Costar, 8.0 µm pore size) with type I collagen as we previously reported [25]. Briefly, cells were harvested with PBS containing 5 mM EDTA, washed, and resuspended in serum-free RPMI1640 media at a concentration of 2–5×10^5^ cells/ml. An aliquot of 100 ul of the cell suspension was added into the upper chamber of the Transwell system. Type I collagen was dissolved in serum-free RPMI1640 media at a concentration of 10 µg/ml and added into the lower chamber of the system. Antibodies (5 µg/ml) or DNA aptamers at indicated concentrations in the text were added in both upper and lower chambers. Migration assays were performed at 37°C for 4–5 hours. The inserts were removed and then fixed in 10% formalin in PBS for five minutes and then stained with 0.5% crystal violet for five minutes. The inserts were then washed and the upper surface of the membranes was wiped with a cotton swab to remove non-migratory cells. Migrated cells were counted in three randomly selected fields. Each experiment was performed in triplicate and repeated three times. The results presented are shown as mean cell numbers +/− S.D. per mm^2^.

### Adhesion Assays

Adhesion of tumor cells to type I collagen was evaluated as described previously with minor modifications [25]. 96-well plates were coated with type I collagen at a concentration of 5 µg/ml and blocked with PBS containing 1 mg/ml BSA. Cells were harvested with PBS containing 5 mM EDTA, washed, resuspended in RPMI1640 containing 1 mg/ml BSA (adhesion buffer) at a concentration of 10^5^ cells/ml. Cells (100 µl/well) were incubated on plates coated with type I collagen in the presence of antibodies at a concentration of 5 µg/ml. Plates were washed with adhesion buffer 4–5 times and remaining cells were fixed followed by staining with crystal violet. After extensive washing to remove excess dye, cells were lysed with 100 µl of PBS containing 2% SDS and then the absorbance was measured at 550 nm. Experiments were performed in quadruplicates and repeated three times. The representative data were shown as mean +/− S.D. of absorbance values.

### Transfections

Full-length CD44E was amplified from cDNA prepared from total RNA of HCC38 by reverse transcriptase reactions. The amplified CD44E was constructed in pIRES2-EGFP. The sequence was verified at WRI. MCF-7 cells were transfected with pIRES2-EGFP alone (vector control) or pIRES2-2-EGFP (CD44E) using Fugene6 as a vehicle. Transfectants were selected by culturing cells in the presence of G418 (100 µg/ml) for 30 days.

### Recombinant Protein

Recombinant exon v10 of CD44 is expressed as a fusion protein with FLAG using pFLAG-CTC vector [26]. The protein was induced to be expressed by culturing cells in the presence of IPTG (1 mg/ml) for 1 hour. Cells were collected and lysed in 100 mM Tris-HCl (pH7.5) containing 1% Triton X-100, 0.14 M NaCl, and protease inhibitors. The lysates were then applied onto a anti-FLAG (M2) antibody-conjugated agarose column, extensively washed with 100 mM Tris-HCl (pH7.5) containing 1% Triton X-100, 1 M NaCl, and protease inhibitors. The bound protein was eluted with 1 M Glycine (pH 2.0) and immediately neutralized with 1M Tris (pH 10). The eluted protein was separated with SDS-PAGE, transferred onto Immobilon-P membrane, and blotted with HRP-conjugated anti-FLAG (M2) antibody to ensure the presence of the fusion protein without degradation.

### Selection of ssDNA that Bind CD44 Exon v10 Peptide

DNA aptamers that bind to the CD44 exon v10 peptide were isolated using buffer systems as described in [1]. Briefly, 96-well plates were first coated with anti-FLAG (M2) antibody at a concentration of 5 µg/ml in PBS and then the non-specific binding sites were blocked by incubating with PBS containing 1 mg/ml BSA for 1 hour at room temperature. Plates were washed with PBS and incubated with CD44 exon v10 peptide for 3 hours at room temperature. 5 nmol of the chemically synthesized ssDNA library dissolved in binding buffer (100 mM NaCl, 5 mM MgCl_2_, pH 7.2) was added (50 µl/well) to the wells coated with recombinant CD44 v10 peptide. The plates were incubated for 1 h at 37°C, and extensively washed with wash buffer (150 mM NaCl, 5 mM MgCl_2_, pH7.2) to remove unbound ssDNA. The bound ssDNAs were recovered by incubating with 30 µl/well of elution buffer (1.5 M NaCl, 5 mM MgCl_2_, pH7.2) for 15 min at room temperature. The recovered ssDNAs (1 µl) were then amplified by unidirectional PCR in favor of the generation of sense strand with 400 pmole of forward primer (5′ - GGGAGACAAGAATAAACGCAA-3′) and 4 pmole of reverse primer (5′-GCCTGTTGTGAGCCTCCTGTCGAA-3′). The amplified aptamers were then incubated on the plates coated with CD44 exon v10 peptide as described above. This selection process was repeated 10 times. After the final selection, bound DNA aptamers were amplified by PCR and ligated to pCR2.1-TOPOTA vector for sequencing.

### FACS Analysis

Expression of integrin subunits was evaluated by FACS analysis according to the methods previously described [27]. Briefly, cells were harvested with PBS containing 5 mM EDTA and immediately neutralized in FACS buffer (RPMI1640 containing 1% BSA and 0.025% NaN3). Tumor cells (10^5^) were incubated with 5 µg/ml of primary antibodies or 5 µg/ml of biotinylated aptamers by shaking for 1 hour at 4°C. After extensive washing with FACS buffer, cells were incubated with FITC-conjugated secondary antibody (1∶1000 dilutions) or FITC-avidin (1∶10,000 dilution) by shaking for 1 hour at 4°C. Cells were then washed with FACS buffer 5 to 6 times and fixed in PBS containing 1% para-fomaldehyde. The experimental conditions were optimized by altering the concentration of antibodies, biotinylated aptamers, and Streptavidin-FITC. Expression was measured on FACS Calibur (Becton-Dickinson, NJ USA) by collecting 10,000 events and analyzed by CellQuest.

### Co-immunoprecipitation Assays

Cells were cultured and washed with PBS briefly to remove serum components [28]. Cells were directly lysed with 100 mM Tris-HCl (pH 7.5) containing 1% Brij35, 0.14 M NaCl, 1 mM CaCl_2_, 1 mM MnCl_2_, and a protease inhibitor cocktail by scraping and pipetting. Cell lysates were centrifuged and the supernatant was precleared with control IgG-conjugated protein G-agarose beads. The precleared cell lysates were immunoprecipitated with anti-CD44 antibody-protein G beads or control IgG-protein G beads for 4 hours at 4°C. The beads were extensively washed and the bound proteins were released by boiling at 95°C in SDS-sample buffer under reducing conditions and separated on SDS-PAGE followed by western blotting with anti-EphA2 antibody followed by HRP-secondary antibody. The proteins are visualized with enhanced chemiluminescence (ECL) reaction.

### Pull-down Assays

Pull-down assays were performed as described previously [27]. Briefly, cells were cultured in dishes (10 cm diameter) overnight in RPMI1640 containing 10% FBS. Cells were harvested using 5 mM EDTA in PBS and were briefly washed three times with PBS. Cell lysates in 100 mM Tris-HCl (pH 7.5) containing 1% Brij35, 0.14 M NaCl, 1 mM CaCl_2_, 1 mM MnCl_2_, and a protease inhibitor cocktail were centrifuged and the supernatant was precleared with anti-FLAG M2 agarose beads (Sigma) for 4 hours by shaking at 4°C. The cleared lysates were incubated with recombinant protein CD44v10-FLAG-anti-FLAG (M2) antibody-agarose beads in the presence or absence of aptamers (20 nM) by shaking at 4°C for 4 hours. The beads were washed extensively with 100 mM Tris-HCl (pH 7.5) containing 1% Brij35, 0.14 M NaCl, 1 mM CaCl_2_, 1 mM MnCl_2_, and a protease inhibitor cocktail to remove any proteins that nonspecifically bound on the beads. The bound proteins were released by boiling at 95°C in SDS-sample buffer under reducing conditions and separated on SDS-PAGE followed by western blotting with anti-EphA2 antibody followed by HRP-secondary antibody. The proteins are visualized with enhanced chemiluminescence (ECL) reaction.

### Statistical Analysis

Two-tailed (paired) Student’s *t*-test was used to calculate statistical significance between control and experimental groups. A *p* value of less than 0.001 is considered as significant difference between the groups.

## Results

### Exon v10 of CD44 Regulated Triple-negative Breast Cancer Migration

**Figure 1:**
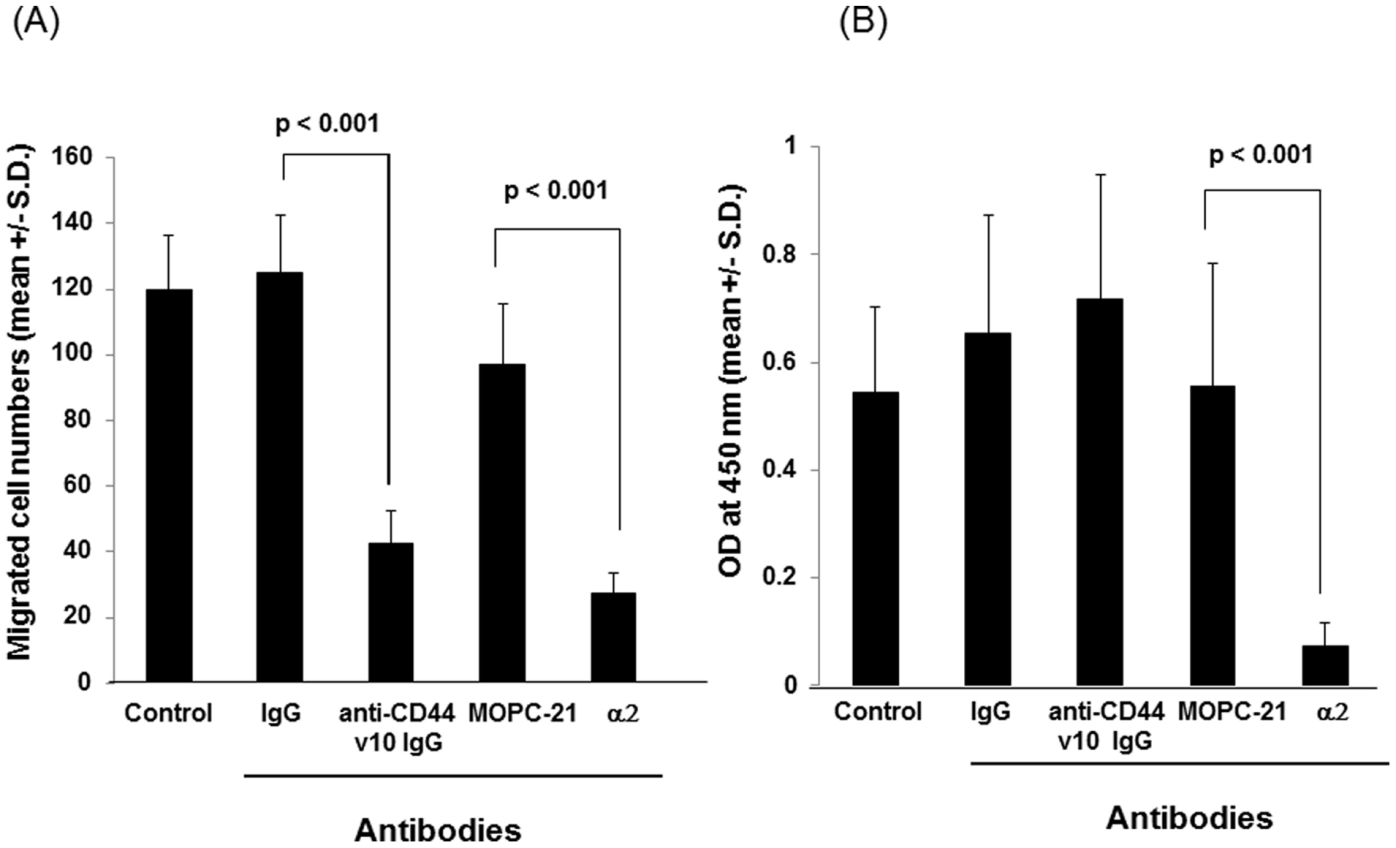
Inhibition of migration of HCC38 cells with anti-CD44 exon v10 antibody. (A) Cells were harvested, washed, and resuspended in RPMI1640-serum free media. Migration assays were performed using type I collagen (10 µg/ml) as an adhesive substrate in the lower compartment of Transwell by incubating at 37°C for 4 hours. The antibodies were added in both upper and lower chambers at a concentration of 5 µg/ml. Experiments were performed by triplicates three times. Statistical significance was calculated by Student’s two-tailed paired *t*-test. (B) Cells were harvested, washed, resuspended in RPMI1640 containing 1 mg/ml BSA (adhesion buffer) at a concentration of 10^5^ cell/ml. Cells (100 µl/well) were incubated on plates coated with type I collagen (5 µg/ml) for 1 hour in the presence or absence of antibodies (5 µg/ml). Plates were washed, fixed followed by staining with crystal violet. After extensive washing to remove excess dye, cells were lysed with 100 µl of PBS containing 2% SDS and then the absorbance was measured at 550 nm. Experiments were performed in quadruplicates and repeated three times. The representative data were shown as mean +/− S.D. of absorbance values.

Enhanced migration is one of the key features of malignant tumor phenotypes. Tumor cells must migrate into connective tissues in order to disseminate from primary tumor for establishing metastasis. Previous studies demonstrated that antibodies against CD44 exon v10 inhibited leukocytes migration to inflammation sites and homing to bone marrow, suggesting that this exon plays a key role in regulating the processes of cell adhesion and migration [22], [23], [24]. To determine if CD44 exon v10 was involved in tumor cell migration, we tested anti-CD44 v10 antibody for its ability to inhibit migration of triple-negative (TN) breast cancer cells, HCC38. Under our experimental conditions, anti-CD44v10 antibody, but not the control antibody, significantly inhibited tumor migration towards type I collagen (Figure 1A), suggesting that this exon regulates TN breast cancer migration. In order to test if the decreased migration was due to the inhibition of cell adhesion to type I collagen, cells were pre-incubated with control IgG or anti-CD44 exon v10 antibody. In contrast to cell migration, the anti-CD44 exon v10 antibody did not affect cell adhesion (Figure 1B). Consistent with our previous studies [25], TN breast cancer adhesion and migration to type I collagen were significantly inhibited by anti-α2 integrin antibody (Figures 1A and 1B). Since HCC38 cell adhesion to type I collagen is mediated by α2β1 integrin these results suggest that CD44 exon v10 is not involved in cell adhesion; it rather functions in post-adhesion processes such as regulating signaling pathways.

### Selection of DNA Aptamers that Bind CD44 Exon v10 Peptide

**Figure 2:**
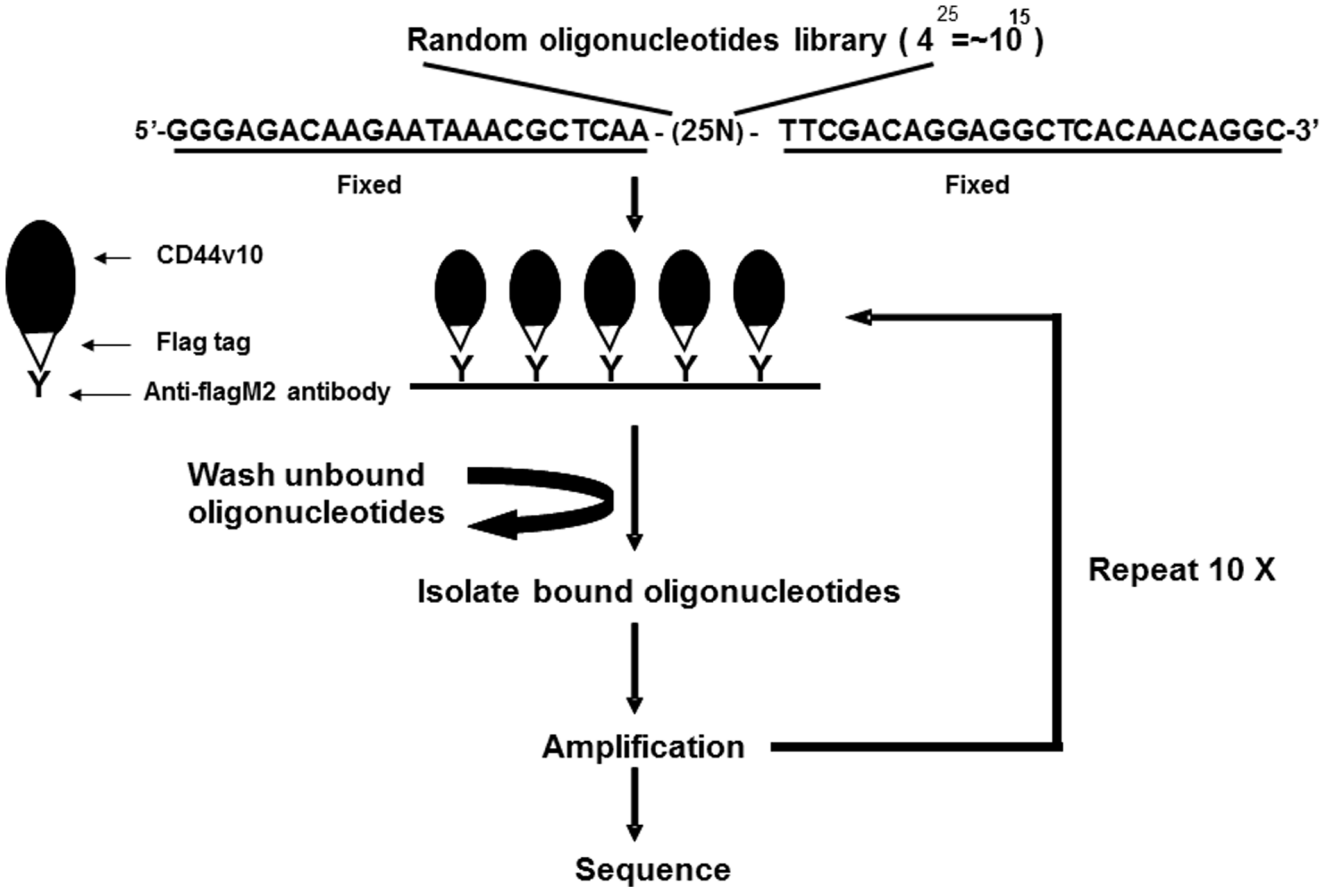
Protocol for isolating CD44 exon v10-specific DNA aptamers by Systematic Evolution of Ligands by Exponential Enrichment (SELEX). The CD44 exon v10 peptide was expressed as a fusion protein with FLAG tag. The purified recombinant peptide was immobilized on plates coated with anti-FLAG (M2) antibody. Library of DNA aptamers were incubated on the plates and extensively washed to remove non-bound aptamers. The isolated aptamers were then amplified and this selection process was repeated 10 times. Aptamers isolated from the last selection were amplified and ligated to pCR2.1-TOPOTA vector for sequencing.

In order to establish screening systems to isolate target specific DNA aptamers, we expressed exon v10 as a recombinant fusion protein with FLAG using pFLAG-CTC in E.coli DE3(BL21) Plys according to methods described in materials and methods. The expressed FLAG-CD44 v10 was immobilized on anti-FLAG (M2) antibody-coated plates and non specific binding sites were blocked by incubating with PBS-BSA, which served as a matrix to isolate and purify DNA aptamers that bind to exon v10 (Figure 2). The first synthetic ssDNA library was generated by 25 random nucleotides flanked by 5′-fixed oligonucleotides (GGGAGACAAGAATAAACGCAA) and 3′-fixed oligonucleotides (TTCGACAGGAGGCTCACAACAGGC) as described previously [1], [12]. This size (4^25^ = ∼10^15^) was selected because it has been reported as the theoretical minima to obtain full library diversity and allow formation of all secondary structures known for ssDNA oligonucleotides as described previously [1].

ssDNA library dissolved in binding buffer was incubated on the plates coated with CD44 exon v10 peptide for 1 h at 37°C. After extensive washing of the plates with wash buffer, ssDNA that bound to the target molecule were recovered by incubating with dissociation buffer. The recovered ssDNA were amplified by unidirectional PCR reactions to enrich sense ssDNA and then incubated on the plates coated with recombinant CD44 exon v10 as described above. We repeated this selection cycle 10 times. At the end of the final cycle of the selection, aptamers that bind to CD44 exon v10 of were isolated, amplified by PCR, and ligated into pCR2.1-TOPO^TA^ vector for sequencing.

### Characterization of Exon v10 Aptamers by FACS Analysis

**Figure 3:**
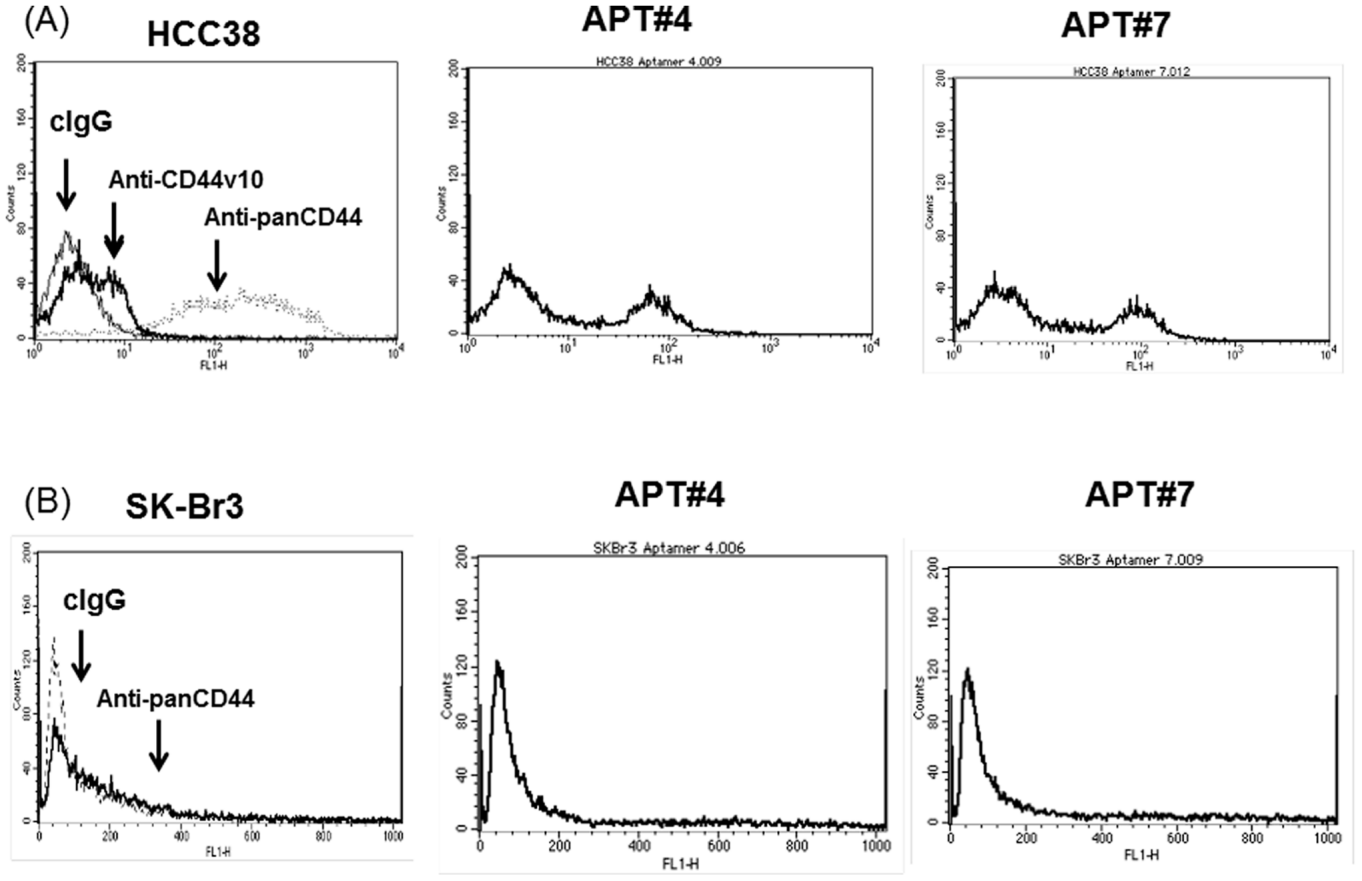
FACS analysis of aptamers against CD44 exon v10. HCC38 or SK-Br3 cells were harvested and resuspended in RPMI1640 containing 1 mg/ml BSA and 0.025% NaN_3_ (FACS buffer). Cells were incubated with aptamers of which 5′-end was conjugated to biotin (5 µg/ml), anti-panCD44 (clone 156-3C11) (5 µg/ml), or anti-CD44 exon v10 antibody (AB2082) (5 µg/ml) for 1 hour at 4°C. After washing with the same buffer, cells were incubated with Streptavidin-FITC, FITC-goat anti-mouse IgG, or FITC-goat anti-rabbit IgG for 1 hour at 4°C. After extensive washing with FACS buffer, cells were resuspended in PBS containing 1% paraformaldehyde. Expression was measured on FACS Calibur (Becton-Dickinson, NJ USA) by collecting 10,000 events and analyzed by CellQuest.

In order to further characterize the selected aptamers for recognizing CD44 expressed on HCC38 cells, the 5′-terminal of each aptamer was conjugated to biotin and then incubated with cells followed by incubation with FITC-avidin. The binding of the aptamers on cell surface was analyzed on FACScalibur. We isolated two aptamers, Apt#4 (GGGAGACAAGAATAAACGCAA CTC CCA GCC CCT CAC GTC AGC CCG C TTCGACAGGAGGCTCACAACAGGC ) and Apt#7 (GGGAGACAAGAATAAACGCAA CCG CGA ACC CCC CCC CTT AAT GTC A TTCGACAGGAGGCTCACAACAGGC ) that recognized HCC38 cells expressing CD44 (Figure 3A). Although nearly all cells were positive to anti-panCD44 antibody (156-3C11), only a fraction of cells were stained with anti-CD44 v10 antibody (Figure 3A), suggesting HCC38 cells express various isoforms of CD44 and exon v10. This notion was consistent with the recent studies demonstrating that cultured human breast cancer cells shows heterogeneity of the expression of various isoforms of CD44 [29]. Under our experimental conditions, Apt#4 and Apt#7 stained around 50% of HCC38 cells (Figure 3A). In order to further test the specificities of aptamers, SK-Br-3 cells, which express low level of endogenous CD44 molecules [30], were incubated with Apt#4 and Apt#7 followed by FITC-avidin. None of the aptamers stained SK-Br-3 cells (Figure 3B), suggesting that the DNA aptamers recognize CD44 exon v10 on the tumor cell surface of HCC38 cells.

### Inhibition of Tumor Migration by Aptamers for Exon v10 of CD44

**Figure 4:**
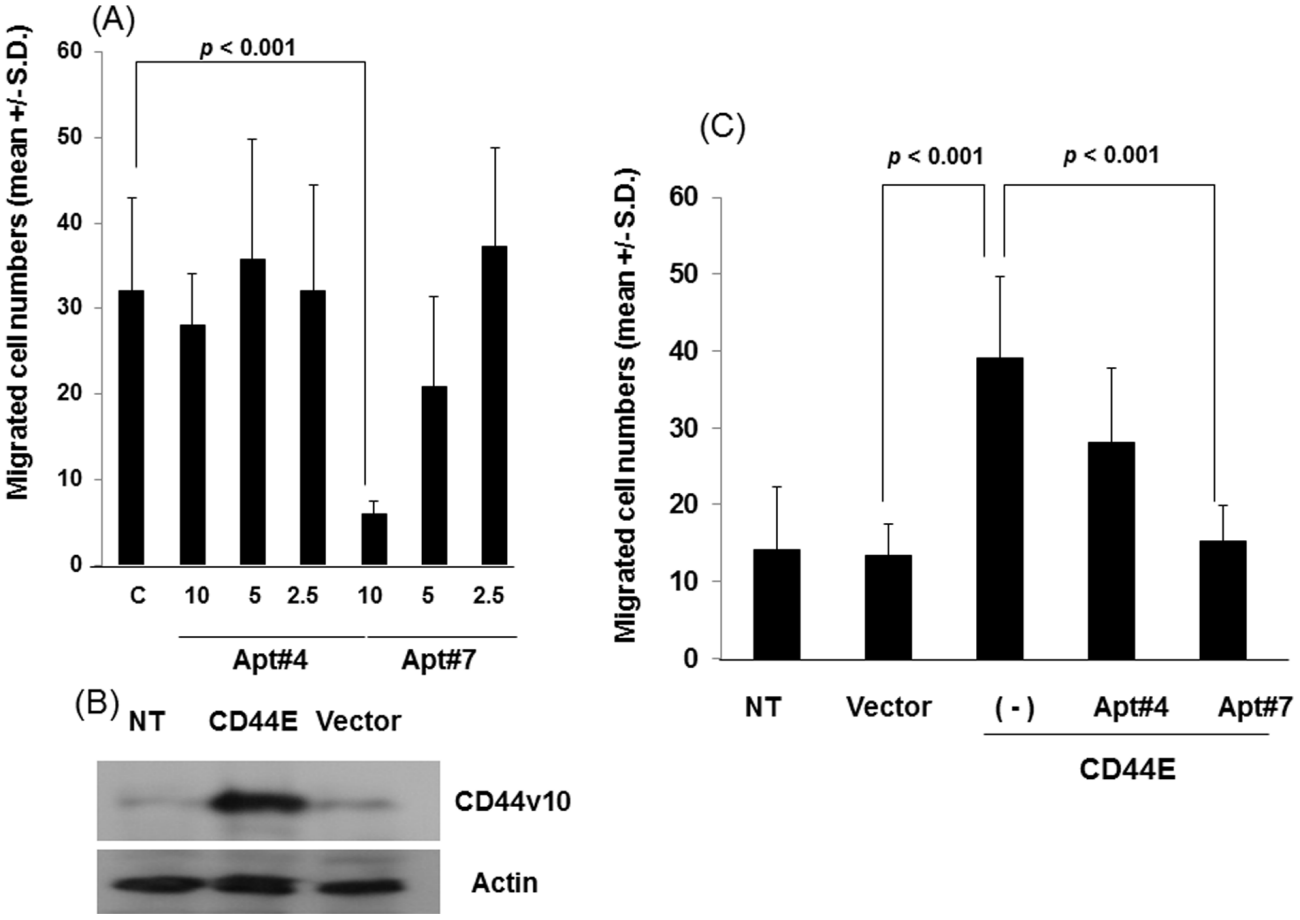
Inhibition of cell migration by aptamers against CD44 exon v10. (A) HCC38 cells were harvested, washed and resuspended in RPMI1640-serum free media. Migration assays were performed using type I collagen (10 µg/ml) as an adhesive substrate in the lower compartment of Transwell by incubating at 37°C for 4 hours. The aptamers were added in both upper and lower chambers at the indicated concentrations in the text. Experiments were performed in triplicate. Statistical significance was calculated by Student’s two-tailed paired *t*-test. (B) MCF-7 cells were stably transfected with CD44E according to the methods described in Materials and Methods. MCF-7 cells transfected control vector, [MCF-7(pIRES2)], MCF-7 (CD44E), and parental MCF-7 cells (NT) were lysed and subjected to western blotting for the expression of CD44E using anti-CD44 exon v10 antibody followed by HRP-conjugated goat-anti-rabbit IgG. The signal was detected by ECL reactions. Actin was used as a loading control. (C) The migration of these cells was evaluated as described above in the presence or absence of aptamers against CD44 exon v10 at a concentration of 10 nM as described above. Experiments were performed in triplicate. Statistical significance was calculated by Student’s two-tailed paired *t*-test.

We then evaluated Apt#4 and Apt#7 for their ability to inhibit tumor migration toward type I collagen as described in the Materials and Methods section. Apt#7 inhibited tumor migration in a concentration dependent manner and significant inhibition was observed at a concentration of 10 nM (Figure 4A), of which concentration was similar to that DNA aptamers to inhibit breast cancer migration [7]. However, Apt#4 failed to inhibit migration despite the fact that this aptamer recognizes HCC38 cell surface (Figure 4A). Thus, we utilized Apt#4 as a negative control for Apt#7 in the following studies.

In order to further test the ability of aptamers to inhibit migration of breast cancer cells, we generated a stable transfectant of CD44E using MCF-7 as a parental cell line. Although MCF-7 expressed CD44 harboring exon v10 as previously reported [31], transfection of CD44E cDNA augmented the expression of the exon as demonstrated by western blotting (Figure 4B). Previous studies demonstrated that transfection of CD44 variant that harbor exon v10 into MCF-7 also augmented migration [19]. Consistent with these results, overexpression of CD44E in MCF-7 significantly enhanced migration to type I collagen (Figure 4B). Importantly, Apt#7 but not Apt#4 significantly inhibited the enhanced migration at a concentration of 10 nM (Figure 4C), which was an optimized concentration in Figure 4A. These results further support a notion of CD44E as a migration-promoting molecule and the aptamers against CD44 exon v10 also functioned as an inhibitor for migration of CD44E-overexpressing cells.

### Association of EphA2 with CD44 on HCC38 Breast Cancer Cells

**Figure 5:**
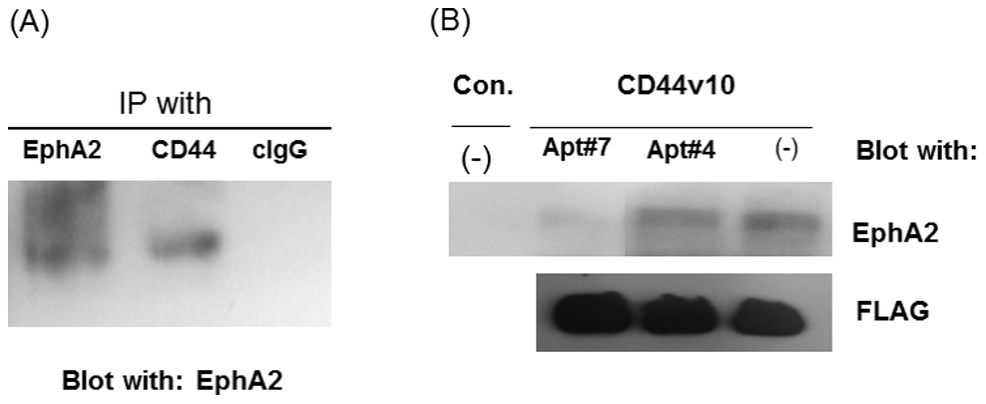
CD44 associated with EphA2 on breast cancer cells. (A) HCC38 cells were lysed with 100 mM Tris-HCl (pH 7.5) containing 1% Brij35, 0.14 M NaCl, 1 mM CaCl_2_, 1 mM MnCl_2_, and a protease inhibitor cocktail by scraping and pipetting. The cell lysates were precleared and immunoprecipitated with anti-CD44 antibody clone (clone 156-3C11)-, anti-EphA2- or control IgG-protein G beads for 4 hours at 4°C. The bound proteins were released by boiling at 95°C in SDS-sample buffer under reducing conditions and separated on SDS-PAGE followed by western blotting with anti-EphA2 antibody followed by HRP-secondary antibody. The proteins are visualized with enhanced chemiluminescence (ECL) reaction. (B) Cells were harvested using 5 mM EDTA in PBS and were lysed in 100 mM Tris-HCL (pH 7.5) containing 1% Brij35, 0.14 M NaCl, 1 mM CaCl_2_, 1 mM MnCl_2_, and a protease inhibitor cocktail. The lysates were precleared with anti-FLAG (M2) agarose beads for 4 hours by shaking at 4°C. The cleared lysates were incubated with recombinant protein CD44v10 P-FLAG-anti-FLAG (M2) antibody-agarose beads (CD44v10) or anti-FLAG (M2) antibody-agarose beads (Con.) in the presence or absence of aptamers (Apt#4 and Apt#7) by shaking at 4°C for 4 hours. The beads were washed extensively with the lysis buffer. The bound proteins were released by boiling at 95°C in SDS-sample buffer under reducing conditions and separated on SDS-PAGE followed by western blotting with anti-EphA2 followed by HRP-secondary antibody or HRP-conjugated anti-FLAG (M2) antibody. The proteins are visualized with enhanced chemiluminescence (ECL) reaction. CD44 exon v10 peptide was used as a loading control for ensuring the same amount of proteins was loaded in each lane.

Since exon v10 localizes in the ectodomain of CD44 and reagents such as antibodies and DNA aptamers inhibited migration of HCC38 cells, we hypothesized that CD44 forms a molecular complex with other cell surface molecules which promotes migration of TN breast cancer cells. In order to identify molecules associated with CD44, we performed co-immunoprecipitation studies in HCC38 cells. Cell lysates were immunoprecipitated with control IgG or anti-CD44 antibody (clone 156-3C11), and the precipitates were separated on SDS-PAGE. The membranes were blotted with antibodies against various cell surface molecules, and we identified EphA2 as one of the molecules that associates with CD44 (Figure 5A). In order to test whether exon v10 of CD44 is directly associated with EpHA2, we performed pull-down assays using the recombinant fragment of CD44 exonv10 as a matrix. Under our experimental conditions, EphA2 was precipitated with the recombinant fragment (Figure 5B), suggesting that EphA2 associates with CD44 exon v10. Importantly, this association was prevented in the presence of inhibitory aptamer Apt#7 but not with non-inhibitory aptamer Apt#4 (Figure 5B). Given the fact that EphA2 regulates tumor cell migration and invasion [32], [33], [34], our results suggest that it forms a molecular complex with CD44 through exon v10 and this promotes TN breast cancer migration. Thus destruction of the interaction using DNA aptamers against the exon would inhibit these processes.

**Figure 6:**
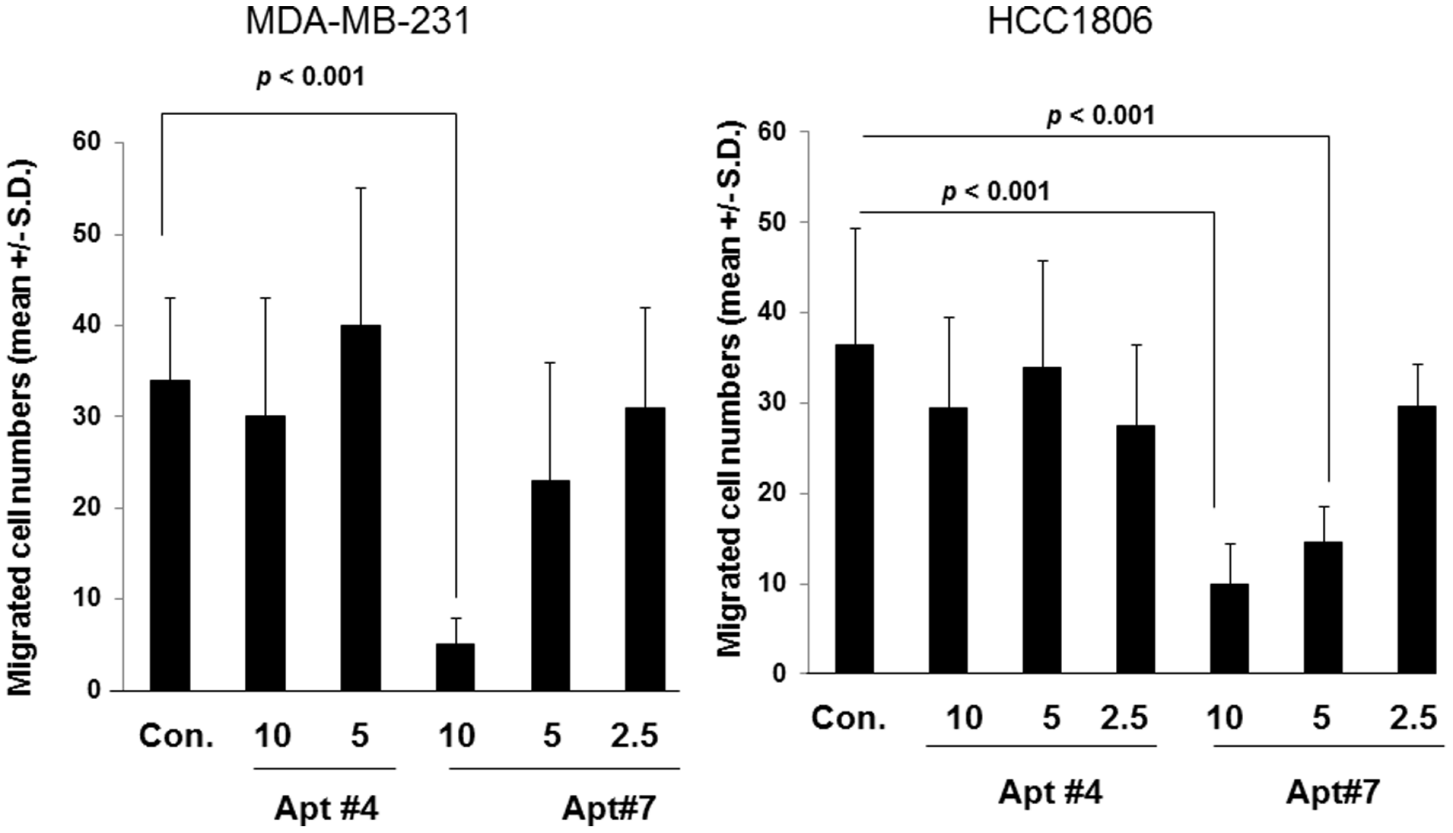
Inhibition of migration of MDA-MB-231 and HCC1806. MDA-MB-231 (A) or HCC1806 (B) cells were harvested, washed and resuspended in RPMI1640-serum free media. Migration assays were performed using type I collagen (10 µg/ml) as an adhesive substrate in the lower compartment of Transwell by incubating at 37°C for 4 hours. The aptamers were added in both upper and lower chambers at the indicated concentrations. Experiments were performed in triplicate. Statistical significance was calculated by Student’s two-tailed paired *t*-test.

We also tested these aptamers for inhibiting cell migration in other cell lines, MDA-MB-231 and HCC1806 cells, which have demonstrated to express EphA2, and we confirmed the expression by western blotting analysis (not shown) [35], [36]. Migration of both MDA-MB-231 and HCC1806 cells toward type I collagen was inhibited in the presence of Apt#7 in a concentration-dependent manner, however Apt#4 did not inhibit migration at any concentrations (Figure 6A and Figure 6B), consistent with the previous results (Figure 4A). These results suggest that Apt#7 would inhibit cell migration expressing CD44 exon v10 and EphA2 by preventing the association of these molecules on cancer cell surface.

## Discussion

Recent studies suggest that breast cancer cells express various CD44 isoforms [29]. For example, Luminal A breast cancer cells show significantly higher expression of CD44v2-v10 and v3-v10 isoforms compared to other subtypes, and basal- normal-type breast cancer cells express CD44E isoforms [29]. Pathological studies showed that the expression of specific exons such as v3, v6, and v9 isoforms is correlated with breast cancer progression [37], [38]. Recent studies demonstrated that hypoxia regulates specific variants (i.e. v6 and v7/8) of CD44 through HIF-1α in triple-negative breast cancer cells [39]. Although the details of the functions of each exon in breast cancer biology are not clear, these results suggest that the functional diversity of CD44 would be due to the structural diversity of the molecule as a result of splicing by tumor-host microenvironments. We demonstrated that EphA2 was co-precipitated with CD44 and was pulled down by exon10 peptide-conjugated beads, suggesting that CD44 forms a molecular complex with EphA2 through exon v10 on breast cancer cell surface. EphA2 is a receptor tyrosine kinase for Ephrin1 and plays a key role in promoting progression and metastasis of various tumor cells including breast cancer [34], [40], [41], [42]. Our results suggest a novel model of molecular complex consisting of CD44 and EphA2 that promote of tumor progression and metastasis. However, there are still questions regarding the mechanisms of the formation of the molecular complex; whether the complex is pre-formed on the cell surface or the ECM-ligand induces the formation on the cancer cell surface, which is a key question for understanding the signaling pathways that promote cell migration.

Aptamers have been demonstrated as alternatives to antibody-based diagnosis, research tools, diagnosis, and therapies [43]. We developed an aptamer, Apt#7, as a potent inhibitor for migration of various breast cancer cells including HCC38, HCC1806, and MDA-MB-231. Significant inhibition was observed at 5–10 nM of Apt#7 dependent on cell types, a concentration that is comparable for that of DNA aptamers against osteopontin to inhibit migration, invasion, as well as tyrosine kinase signaling pathways [7]. The fact that the association of EphA2 and CD44 exon v10 was not observed in the presence of Apt#7 suggest that one of the mechanisms would be that this aptamer prevents the association of the molecular complex consisting of CD44 and EphA2 on cancer cell surface. Despite the fact that both Apt#7 and Apt#4 recognized HCC38 cell surface, Apt#4 failed to show any inhibitory activities in migration and CD44 exon v10 interaction with EphA2. Although the reasons to explain the inactivity of Apt#4 are not clear, it is possible that Apt#7 and Apt#4 would bind distinct site of CD44 exon v10 peptide. The ability of these aptamers to recognize amino acid residues in CD44 exon v10 would be particularly valuable where antibodies cannot recognize. Indeed, previous studies demonstrated the efficacy of mutation analysis of target proteins to identify specific aptamer binding sites [44], [45]. It is expected that the identification of Apt#4 and Apt#7 would provide significant information to characterize the nature of the interaction between CD44 exon v10 and EphA2. Since we cannot exclude the possibility that the affinity of Apt#4 to CD44 exon v10 would be lower compared to that of Apt#7, we are evaluating dissociation constants between these aptamers and CD44 exon v10 peptide and its mutants.

In this study, we developed DNA aptamer that bind to CD44 exon v10 and demonstrated the potential role for CD44-EphA2 complex to promote breast cancer migration. Recent studies by Gorenstein and colleagues developed DNA aptamer against hyaluronan (HA) binding site (HABD) of CD44 and demonstrated the efficacy to inhibit the interaction of HA to CD44 [46]. Given the fact that HCC38, MDA-MB-231, and HCC1806 are classified in distinct subtypes of TN breast cancer cells [47], the results of inhibition of migration by attenuating CD44 exon v10 function may lead to a promising approach for inhibiting tumor progression and metastasis of any TN breast cancer cell types.
